# Driving factors and grouping paths of rural sports development in China ‐ A qualitative comparative analysis based on fuzzy sets

**DOI:** 10.1371/journal.pone.0300169

**Published:** 2024-03-28

**Authors:** Duan Yu, Hongwei Fan, Ning Zhang

**Affiliations:** 1 Institute of Sport Training, Chengdu Sport University, Chengdu, China; 2 School of Economics and Law, Southwest University of Political Science and Law, Chongqing, China; IBS Hyderabad: ICFAI Business School, INDIA

## Abstract

The development of rural sports depends on many factors, but the complex causal relationship between various factors and the level of rural sports development is not clear. Using data envelopment analysis (DEA) and fuzzy set qualitative comparative analysis (fsQCA), this study aims to examine the driving role of various factors on rural sports development and construct various grouping paths to improve the level of rural sports development in China. The results shows that the area of fitness venues and social capital participation are sufficient conditions for the development of rural sports in China. Resource endowment through government fing, social capital participation and the construction of sports venues and facilities is the key driving factor for rural sports development in China. There are four grouping paths for the high-quality development of rural sports, which are divided into three configurations by combining the grouping characteristics: the state-social capital jointly controlled type driven by economic development and the resource endowment driven by the modernization of the agriculture promotes production and the grassroots organizations that are supported by the advantage of resource endowment. The results of the study highlight the complex causal relationships and key driving factors of rural sports development in China, highlight the differences in rural sports development patterns in different regions, and provide new ideas and guidelines for improving the level and quality of rural sports development.

## 1. Introduction

The Guiding Opinions on Promoting the High-Quality Development of Peasant Sports in the Fourteenth Five-Year Plan clearly state that the development of rural sports is an important task for comprehensively promoting rural revitalization, building a strong sports nation and a healthy China. It is necessary to continuously meet the needs of rural people for a better life, improve and perfect the public service system for farmers’ fitness, promote the high-quality development of farmers’ sports and fitness enterprises, and then a rural environment to create sports development patterns with the diverse and deep integration of agricultural sports, culture and sports, and wisdom and sports [1]. By 2023, China’s rural resident population has reached 564.01 million. The development of rural sports is not only a necessity to ensure that more than 500 million farmers in China enjoy basic sports rights and interests and basic sports services, but also an indispensable and important part of the comprehensive rural revitalization [2]. The development of rural sports plays an important role in increasing the physical activity levels of rural residents, promoting their physical and mental health and strengthening social cohesion [3].

Thanks to the comprehensive promotion of the rural revitalization strategy and the implementation of several supporting projects. China’s rural sports has made great progress in terms of development mode, development speed and development scope, and is in the transition period from development quantity to development quality [4]. However, due to the sectional sports governance model and the historical structure of the urban-rural dualistic society, rural sports have always been difficult to eliminate the dilemmas and vulnerabilities of a single power source, a single supply organ and the historical dependence on the development path. and an unbalanced economic base [5]. In a study on satisfaction with public sports in urban and rural China, Guo found that rural residents’ satisfaction with public sports is significantly low [6]. The satisfaction gap is primarily reflected in sports advice. As for regional differences, the satisfaction gap between urban and rural public sports facilities is smaller in the eastern provinces than in the central and western provinces. In 2022, there are still more than 50,000 administrative villages in China without sports facilities. The coverage of public sports facilities in rural areas is completely inadequate and more than 50% of the rural population does not take advantage of the services offered by public sports facilities [7]. All signs point to China’s rural sports development level being low. It is difficult to break the shackles of one’s own development in a short period of time, and there is an urgent need for a set of reasonable development models to guide development.

At present, research on rural sports development mainly focuses on the governance logic, supply mode and influencing factors of public services in rural sports. And the research paradigm is dominated by theoretical research, with fewer empirical studies.

In the exploration of rural sports governance logic. The unbalanced economic base, the sole source of authority, the lack of subject participation and the unidirectional operation process have become difficulties in the development of rural sports [8]. As a positive attempt, collaborative governance reconstructs the roles, functions and mechanisms of government, market and social organizations, promotes the transformation of governance issues into diversified issues, and optimizes the effectiveness of rural sports governance while reducing the government’s dominant position in guaranteed by governance [9]. Under the dilemma of insufficient governance target ability, content supply fragmentation and spatial fragmentation of public sports service resources, proposing a precise governance path helps to accurately identify the sports ambitions of rural residents, accurately design special policies for governance and carefully provide public sports services [10]. In rural areas where resources and power are highly dispersed, embedding the theory and methodology of “meta-governance” refines government responsibility and promotes the standardization of power and resources [11].

Nowadays, the provision of public services in rural sports still consists of the rigidity of the supply system and mechanism, the supply subject and the individual supply product, the supply of homogenization and other practical problems [12]. Among them, efficiency priority, standard orientation and supra-regional vision are the main characteristics of public sports provision in rural areas [13]. The optimization of rural public sports provision should take the actual sports needs of rural residents as the starting point, improve the rationality of the supply capital allocation structure, and increase the kinetic energy of social capital supply participation, so as to achieve the precision of rural public sports service provision [14]. In terms of innovation, Yao Lei suggests that a networked supply platform and a multi-layer supply mechanism should be built with the synergistic linkage of multiple actors from government, market and social organizations to form a new pattern of service governance for the integrated development of urban and rural areas [15]. Based on the problem of supply rather than demand, Liu Hongliang believes that farmers’ sports demand should be the logical starting point, and the necessary mechanism to communicate farmers’ sports demand should be established to form a diversified rural sports audience service supply framework with interaction between Supply and demand [16]. On the other hand, the flat governance model proposed by Wang Kai focuses on asking for people’s needs by building an operational system of flattened communication, flattened decision-making, flattened implementation and flattened monitoring [17]. In addition, strengthening government functions, improving resource supply, expanding the majority of participation, and enriching the talent team are also considered the keyways to improve public services of rural sports in China [18].

Existing studies have mainly examined the various factors driving rural sports development at a theoretical level. They are divided into two categories: direct and indirect drivers. So-called direct driving factors are those factors that can have a direct influence on the development of rural sports activities. These include sports venues and facilities in rural areas, sports advisory organizations, rural residents’ knowledge and awareness of physical activity, and sports financing [19]. The absence of all the above elements will result in a limited level of rural sports development. Of the 850,000 sports venues currently owned by China, only 8.18 percent are spread across townships (cities) and villages. The insufficient quantity, poor management and irrational distribution of sports infrastructure in rural areas have limited rural residents’ interest in sports participation to a certain extent [20]. Chen’s study found that building public sports facilities in rural areas can significantly increase the sports participation of rural residents [21]. Added to this are the lack of sports advisory organizations in rural areas, the lack of awareness of physical exercise among rural residents and the insufficient consumption capacity of sporting goods [22]. They will all have varying degrees of impact on the development of rural sports.

Rural sport as a type of sporting activity within the rural area and with the rural population as the main actor involves the development and construction of rural areas as well as the individual development of rural residents [23]. Therefore, the indirect driving factors mainly refer to the factors that can promote the development of rural areas and the individual development of rural residents, and indirectly provide space and conditions for the development of rural sports. These include factors such as the number of rural populations, the level of economic development, infrastructure development and agricultural production methods. Let’s take the number of rural population as an example: if the surplus rural labor flows to the cities, which are superior to rural resources, this leads to a “hollowing out” of rural sports development [24]. The lack of the main force is the fundamental factor limiting the development of rural sports in China. Relevant research shows that rural residents’ awareness of physical activity is directly related to educational level. And in rural areas with better levels of economic development, the educational level of rural residents is often higher [25]. Greater awareness of physical exercise inevitably promotes the development of rural sports. The driving effect of rural infrastructure construction and agricultural production methods on the development of rural sports is mainly reflected in the reduction of production costs, increase of productivity, reduction of working time and higher income of rural residents [26]. Improved infrastructure construction can promote the urbanization of rural areas and provide rural residents with an ecological and livable living environment [27]. Modern agricultural production methods can free rural residents from heavy agricultural activities, improve productivity and increase income [28]. This creates conditions for rural residents to participate in sporting activities.

In the latest study on the development of rural areas, it is shown that the development of new energy in rural areas of China is currently progressing well and that the new energy sector is experiencing overall growth [29]. However, the problems of lagging behind in the development of new energy production equipment and insufficient cleanliness in energy consumption remain. Compared with the target requirements of agricultural and rural modernization, there is still a big gap [30]. Reasonable and effective incentive mechanisms with observable and quantifiable performance indicators will be more favorable to the development of China’s new energy industry in rural areas [31]. In addition, the important role of digital technology in the development of rural areas cannot be ignored. In the context of carbon peaking and carbon neutrality, investing heavily in green innovation projects in rural areas to improve green competitiveness and increase profits can effectively promote the green and rapid development of rural areas [32].

Previous studies have primarily researched the independent influence mechanisms of individual factors on rural sports development and a lot of research has been carried out on the theoretical foundations and development mechanisms of rural sports development. However, the possible connections and group effects between different factors influencing rural sports development have been neglected. Furthermore, there is a lack of systematic study of the pattern characteristics, geographical differences and driving factors of rural sports development in different provinces of China. In summary, the development of rural sports in China is facing many problems, and how to accurately identify the driving factors of rural sports development and build a group path for the high-quality development of rural sports has become an urgent problem. Accordingly, the main purpose of this study is put forward: how can the high-quality development of rural sports be realized? At the same time, it is further decomposed into the following three questions: (1) What are the driving factors of rural sports development? (2) What are the paths of each driving factor to the high-quality development of rural sports? (3) How can the high-quality development of rural sports in different regions be realized, taking into account the various patterns of rural sports development? In view of this, this paper places rural sports development in the spatial heterogeneity domain and constructs a theoretical analysis framework with the development subject, development environment and resource endowment as the set. Using the fuzzy set qualitative comparative analysis method, we explore the role mechanism and linkage relationship of the driving elements of rural sports development from the group perspective.

The novelty of this study is mainly reflected in the following aspects: (1) Through a systematic review of the research literature, a theoretical model of rural sports development was constructed, expanding the scope of exploration of rural sports development issues. (2) Introducing a new research perspective, placing the issue of rural sports development in the theory of spatial heterogeneity. And exploring the role of the development environment, the development entity and the resource endowment on the driving role of rural sports development. Enrich and improve the conclusions of existing research from the theoretical level, and providing a theoretical basis for the development of rural sports. (3) Synthesizing empirical research methods that combine qualitative and quantitative analysis. The super efficiency of China’s rural sports development is evaluated by using an empirical research method combining DEA and fsQCA, analyzing in depth the core and marginal elements of rural sports development, further exploring the driving effect of the combination effect between different elements on the development of rural sports in China. And constructing the paths of different groupings for the development of rural sports in China. The identification of core driving elements and the construction of developmental patterns will also provide more targeted practical guidance for the development of rural sports in China.

## 2. Theoretical basis and analytical framework

### 2.1 Spatial heterogeneity theory

The existence of spatial heterogeneity is an important basis for the development of spatial economics, which focuses on agglomeration theory, and spatial class division and spatial selection as the latest frontier of agglomeration theory explain the unequal distribution of economic activities and the development of things in space from a new perspective [33]. The theory of spatial heterogeneity states that space is a collection of specific resource endowments and economic and social development environments, and that the diversity and complexity of spatial elements constitute the diversity and complexity of space [34]. At the same time, the lack of regional homogeneity in geospace leads to the existence of economic and geographical structures such as developed and underdeveloped regions, centers and peripheral regions, which is the fundamental cause of the differences in the development of things in different spatial locations [35]. As an important part of rural development, the discussion of rural sports development needs to be placed within the framework of rural development, and the introduction of spatial heterogeneity theory provides an innovative perspective and paradigm for interpreting the problem of rural sports development.

Combined with the theory of spatial heterogeneity, the most intuitive explanation for the differences in rural sports development in different geographical areas is the uneven distribution of economic activities in the spatial location, and this unevenness is reflected in the spatial heterogeneity of resource endowment and development environment and population flow [36]. Firstly, there is the issue of resourcing; The presence of heterogeneity affects the choice of location of the economic entity, which in turn leads to the absorption of several resource elements necessary for the development of rural sports by economically developed regions, which creates the phenomenon of a higher level of development of rural sports explained in economically developed regions. The external heterogeneity created by location differences highlights the driving role of the development environment in the development of things [37]. External heterogeneity mainly concerns resource endowment, accessibility and other first and second natural characteristics (geoclimatic, consumption level, social facilities for production, natural disasters, etc.) [38]. Differences in these aspects are also important determinants of the development of things in different spatial locations; For example, rural areas with a better ecological and human environment and better infrastructure development have a higher level of rural sports development, while areas with a poor ecological environment and natural disasters create external constraints for the development of rural sports. The influence of external heterogeneous metropolitan areas on rural sports development through the location basis is a simple and intuitive phenomenon that plays an important role in explaining the differences in the level of rural sports development in different regions. Accordingly, the issue of rural sports development is placed within the theoretical domain of spatial heterogeneity to examine the role of different resource endowments and development environments in promoting rural sports development.

### 2.2 Analytical framework

**Fig 1:**
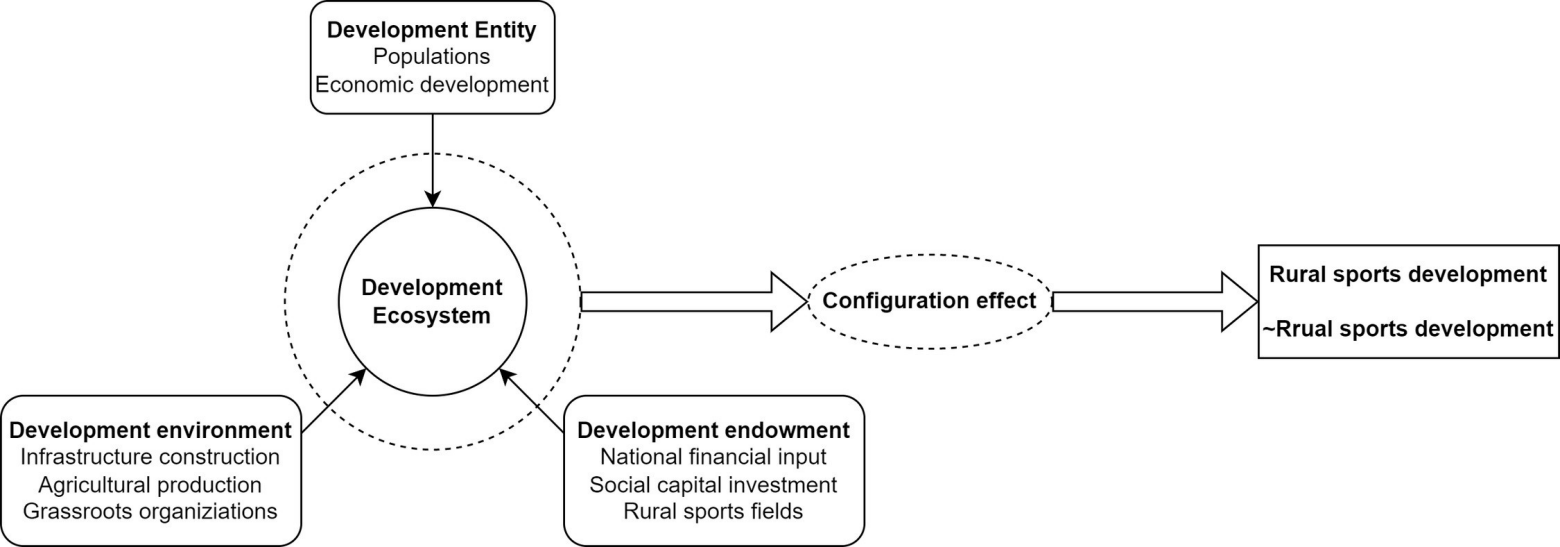
Theoretical model construction of rural sports development.

The diversity and regional heterogeneity of the driving factors of rural sports development also determine their complexity and differentiation. The development of rural sports is essentially a combination of driving factors, and the quality of rural sports development will have greater differences under different combinations of driving factors. Based on the theory of spatial heterogeneity, the driving factors of rural sports development are divided into three main categories: development subject, development environment and resource endowment; A theoretical model of rural sports development is constructed to examine the driving role of rural sports development under the appropriate combination of various factor conditions. The theoretical model of rural sports development created in this study is presented in Fig 1.

In the construction of the theoretical model, the theoretical model of high-quality development of rural sports is mainly composed of three first-level condition variables, namely, development environment, development entity and resource endowment, which form the ecosystem of rural sports development. Under the condition variable of development entity, two secondary condition variables are covered, namely, working-age population and per capita GDP in rural areas. The main purpose is to explore the impact of population and rural economic development on rural sports development. The development environment covers three secondary condition variables. Including infrastructure construction in rural areas, modernization of agricultural production, and the number of rural grassroots sports organizations to explore the role of these three condition variables in driving rural sports development. In resource endowment, the driving roles of state financial input, social capital participation and the construction of sports venues and facilities in different rural areas on rural sports development are mainly analyzed. Finally, under the group effect of different matching combinations of the above condition variables, the development of rural sports in different regions forms two states: high-quality development and nonquality development. In the subsequent introduction of the theoretical model, we analyze in detail the driving effects of development entity, development environment and resource endowment on rural sports development.

#### 2.2.1 Development entity

The driving role of demographic and economic factors in the development of rural sports is obvious. The first and most fundamental issue in the study of rural sports is the structural per capita characteristics of rural sports subjects [39]. As the main force of rural sports development, the working-age population, mainly young adults, has become an important guiding factor for rural sports development. With the acceleration of the urbanization process, the main force of rural sports development continues to shift to the city, and with the loss of the rural sports population, the “hollowing out” of rural sports development is becoming more and more prominent [40]. The driving effect of the main force on the development of rural sports is mainly reflected in the change in the lifestyles and behaviors of rural residents [41]. In contrast to rural populations, young adults in cities often develop healthier and more educated lifestyles and behaviors. Awareness of physical exercise and habits can also be promoted in the family left behind in the countryside, thereby changing the traditional concept of “no disease is healthy” among rural residents [42]. The economic foundation contributes to the development of rural sports. On the one hand, the construction of rural sports facilities in economically developed areas is becoming more precise and diverse, the awareness of good rural habitats and the natural ecological environment is being strengthened, and the awareness of physical exercise among the rural population is constantly improving [43]. On the other hand, higher economic income after meeting basic needs shapes rural residents’ desire for a better life, and participation in sports and exercise activities outside of work activity is one of the symbols [44].

#### 2.2.2 Development environment

The development environment does not directly drive the development of rural sports, but provides opportunities and space for the development of rural sports by improving the rural ecological environment, increasing agricultural productivity and increasing farmers’ income. After using a double difference model to evaluate the relationship between rural infrastructure development and rural residents’ income and their internal mechanisms, the income-increasing effect of rural infrastructure is more obvious in the eastern and central regions, as well as in regions with higher traditional infrastructure levels [45]. In addition, the role of rural infrastructure construction is reflected in promoting the modernization of rural industrial structure, thereby further promoting the quality and expansion of rural residents’ consumption [46]. Regarding the quality of agricultural development, the role of different rural infrastructures in the high-quality development of agriculture is different, with water conservation and communication infrastructure having a significant positive impact on the high-quality development of agriculture [47]. In the process of modernizing agricultural production, mechanized agricultural production methods have improved the productivity of agricultural labor, shortened agricultural working time, increased non-agricultural working time of rural residents, and significantly changed rural lifestyles, creating conditions and opportunities for farmers to participate in sports activities [48]. The grassroots organizations represented by rural cultural stations are an important guarantee for the development of rural sports.

#### 2.2.3 Resource endowment

Combined with the theory of spatial heterogeneity, the amount of resource endowment and the development environment determine the quality of rural sports development. Improved sports facilities with state financial investments and social capital participation are an important guarantee for the development of rural sports, and a variety of sports and fitness facilities increase the motivation of rural residents to take part in sports and exercise activities [49]. In addition, the level of social capital participation is an important indicator of the state of rural sports development in the region. The active participation of social capital reduced the pressure on the state’s financial burden and resource supply. The change in the management role of the government has also created space and opportunities for social and market actors to participate in the development of rural sports, and the formation of a multi-supplier pattern of actors will also contribute to diversifying and improving the quality of rural sports development.

## 3. Research and data methodology

### 3.1 Qualitative comparative analysis and its applicability

Qualitative comparative analysis (QCA) takes Boolean operations and set theory as its methodological basis to explore how combinations of antecedent conditions lead to observable changes or discontinuities in the interpreted results [50], aiming at solving the complex causal relationships between different antecedent conditions and results and at the same time effectively identifying synergistic effects and dynamic complementarities among different factors [51]. In addition, QCA can effectively identify the synergistic effect and dynamic complementarity among different factors, overcoming the limitations of traditional qualitative and quantitative research [52].

Fuzzy set qualitative comparative analysis (fsQCA) is used mainly due to the following considerations: Rural sports development involves the exploration of the complex causality of multiple factors, the different combinations and matches of antecedent conditions will play a role in the results of rural sports development, and the fsQCA method can effectively identify synergies and dynamic complementarities between different factors and can effectively identify synergies and dynamic complementarities between different factors [53]. Compared with the traditional econometric model that insists on the "net effect" analysis of additivity and the assumption of independent variables, the QCA method can analyze the dynamic complementarity and synergistic effect of different antecedents of rural sports development from a holistic perspective, which is helpful for this paper to explore the impact of the linkage and matching of the five antecedents on rural sports development. Second, the QCA method is applicable to both large-sample case studies with more than 100 cases and small- and medium-sample case studies with 10–50 cases, while the 30 cases selected for this paper are medium-size samples, so the QCA method is applicable.

### 3.2 Data envelopment analysis

The data envelopment analysis method is a nonparametric relative efficiency evaluation method based on linear programming and convex analysis, which is applicable to the comprehensive efficiency analysis of decision-making units under the conditions of multiple inputs and multiple outputs [54]. At present, this method has been widely used in the efficiency evaluation of competitive sports and public sports [55, 56]. As the DEA method can flexibly address the relationship between multiple inputs and multiple outputs and does not need to consider the weighted value of the evaluation indices, it greatly reduces the impact of subjective factors in efficiency evaluation [57]. Therefore, this paper adopts the DEA-SBM model in the data envelopment analysis method to measure the development efficiency of rural sports in 30 provinces, autonomous regions and municipalities in China and takes it as the outcome variable for QCA analysis.

#### 3.2.1 Selection of evaluation indicators

The construction of the evaluation index system should follow the principles of accessibility, completeness, operability, and isotropy [58]. Based on relevant studies, the area of farmers’ sports and fitness venues, the number of farmers’ sports and fitness projects, the total amount of investment in farmers’ sports and fitness projects, the number of village and town construction organizations, the number of village and town construction personnel, and the number of cultural stations were selected as input indicators. Rural per capita sports and fitness space area and rural per capita consumption of sporting goods were selected as output indicators. The DEA-SBM model was used to measure the super efficiency value of rural sports development in different regions. The descriptive statistics of the specific input and output indicators are shown in Table 1.

**Table 1:** Descriptive statistical analysis of input and output indicators.

	Variables	Mean	Std	Max	Medium	Min
Inputs	Areas(㎡)	483952.34	711799.31	3691273.75	263382	3000
	Fitness projects	936.37	1176.90	5026	505.50	9
	Fund (100 million)	4297.01	3854.60	12394.61	3005.69	29.00
	Organizations	223.00	171.18	603	198.50	3
	Personnel	649.40	633.14	2904	515.50	18
	Cultural stations	1090.60	694.57	3708	992.50	129
Outputs	Per capita areas	.0258342	.02454660	.10581	.0258342	.00101
	Per capita consumption	1243.04	256.29	1783.8	1238.45	380.1

The use of the DEA method requires a good correlation between the evaluation indicators, i.e., the isotropy requirement. Therefore, SPSS 26.0 software was used to test the correlation of the rural sports development super efficiency evaluation indicators. The test results are shown in Table 2. China’s rural sports development super efficiency evaluation of input and output indicators is at the 1% statistical level through the two-tailed test. This shows that the correlation between the evaluation indicators is good and meets the requirements of the DEA method.

**Table 2:** Results of correlation tests between input and output indicators.

	Areas	Fitness projects	Fund	Organizations	Personnel	Cultural stations	Per capita areas	Per capita consumption
Areas	1.000							
Fitness projects	.962**	1.000						
Fund	.836**	.869**	1.000					
Organizations	.284**	.322**	.266**	1.000				
Personnel	.351**	.407**	.383**	.943**	1.000			
Cultural stations	.713**	.752**	.628**	.703**	.742**	1.000		
Per capita areas	.842**	.721**	.634**	.001**	.026**	.314**	1.000	
Per capita consumption	.316**	.324**	.369**	.224**	.380**	.328**	.086**	1.000

#### 3.2.2 Construction of the DEA-SBM model

Assuming that ρ denotes the efficiency value of DMU (*χ*_0_, *γ*_0_), $s_{k}^{-}\in R_{m}$ denotes the redundancy of the number k’s input, and $s_{r}^{+}\in R_{s}$ denotes the deficiency of the number r’s output. Assuming that there are k inputs and r outputs, k = 1, 2 …, m, and r = 1, 2 …, s, the basic form of the SBM model is as follows:

$$\begin{array}{l} \min\;\rho \text{=}\frac{1-\frac{1}{m}\sum_{k=1}^{m}\frac{s_{k}^{-}}{x_{k0}}}{1+\frac{1}{s}\left(\sum_{r=1}^{s}\frac{s_{r}^{+}}{y_{r0}}\right)}\\ s.t.\;\chi_{0}=\chi \lambda +s^{-},\\ \;\gamma_{0}=\gamma \lambda -s^{+},\\ \;\lambda \geq 0,\;s^{-}\geq 0,\;s^{+}\geq 0, \end{array}$$ (1)

where ρ satisfies 0 < ρ ⩽ 1 and ρ is monotonically decreasing for $s_{k}^{-}$ and $s_{r}^{+}$ when and only when *s*^-^ = *s*^+^ = 0, ρ = 1 and DMU (*x*0,*y*0) is on the efficiency frontier. If a DMU is inefficient, it can be improved to remove slack and thus achieve efficiency improvement.

### 3.3 Selection of variables

#### 3.3.1 Outcome variables

**Fig 2:**
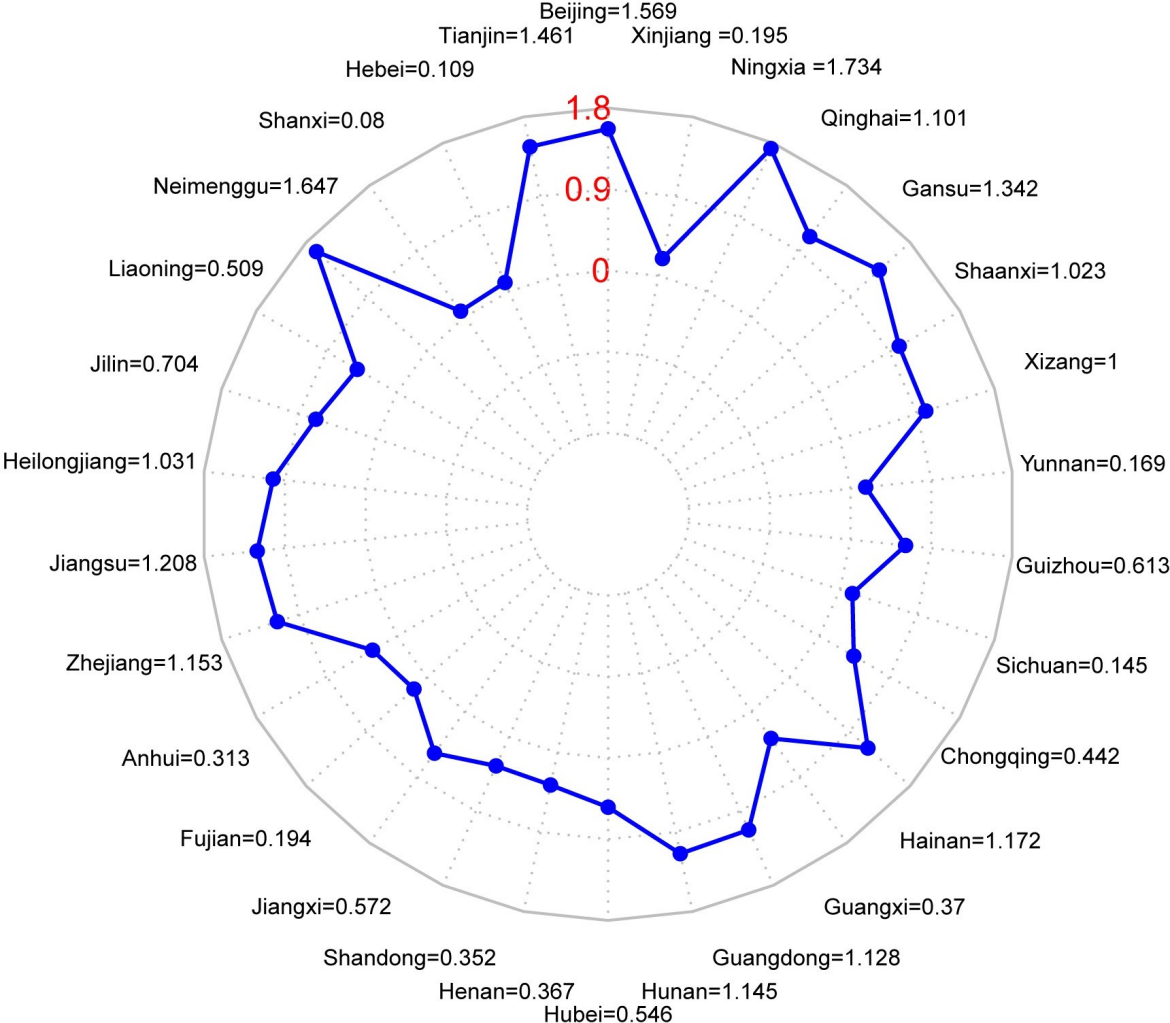
Provincial super-efficiency values of rural sports development in China.

It is difficult for a single variable to reflect the level of rural sports development, and composite indicators are used to make the results of QCA analysis more explanatory. Hence, using data envelopment analysis (DEA), the super efficiency value of rural sports development measured by the DEA-SBM model is taken as the outcome variable and analyzed by QCA. After measuring with DEA-SBM model, the super efficiency of rural sports development in China is shown in Fig 2.

#### 3.3.2 Conditional variables

When applying the QCA method, the balance between the number of conditions and the number of cases must be taken into account. For medium-sized case samples, four to eight preconditions are generally selected. Combined with the principle of selecting preconditions based on the comprehensive consideration of data availability and the completeness of the theoretical framework, eight secondary precondition variables are finally selected, which can be assigned to the three dimensions of development object, development environment and resource endowment. Descriptive statistics for the condition and outcome variables are shown in Table 3.

**Table 3:** Descriptive statistics of conditional and outcome variables.

Variables	Average	St. Error	Min	Max	Size
Working-age population	10640167	6848225	1419716	25331552	30
Per capita disposable income	17244.49	4978.776	10344.3	31930.5	30
Infrastructure development	1161280	953088.5	77367.5	4038929	30
Gross power of agricultural machinery	3517.337	2875.596	120.2	10964.7	30
Number of Cultural stations	1090.6	694.5763	129	3708	30
National financial inputs	2665.513	2919.717	0	11279.65	30
Social capital investment	1631.5	1655.16	0	5552.83	30
Area of rural sports fields	483952.3	711799.3	3000	3691274	30
Sper-efficiency of rural sport	0.779853	0.509569	0.080355	1.734075	30

#### (1) Development entity

Development entities are mainly oriented to indicators related to rural residents and explore the driving role of rural residents in rural sports development. The working-age population in rural areas is selected as the dominant force of rural sports development, and the disposable income per capita of rural residents is selected to represent the level of economic development in rural areas.

#### (2) Development environment

The development environment covers three secondary indicators: infrastructure construction in rural areas, the number of grassroots organizations and the modernization of agricultural production. Among them, rural infrastructure construction is represented by the investment in the construction of rural public facilities, the number of grassroots organizations is represented by the number of village cultural stations, and the modernization of agricultural production is represented by the total power of rural agricultural machinery in each region.

#### (3) Resource endowment

The resources endowment of rural sports development is measured by national financial investment in rural sports and fitness projects, social capital investments and in the area of sports and fitness facilities. The national financial contribution refers to the degree of importance given by the government, the contribution of lottery, public welfare and social funds refers to the degree of social capital participation, and the area of rural sports fields represents the construction of rural sports facilities.

### 3.4 Case selection and data sources

Thirty provinces in China were selected as the case samples for this study (Shanghai, Hong Kong, Macao and Taiwan were not involved in this study due to missing data). On the basis of ensuring the availability and operationalization of the research data, sufficient homogeneity of the case samples and large heterogeneity within the overall cases are fully ensured.

All data in this study are from China Statistical Yearbook of Sports 2020, China Rural Statistical Yearbook 2021, China Statistical Yearbook of Population and Employment 2021 and China Statistical Yearbook of Urban and Rural Construction 2021.

## 4. Results

### 4.1 Variable calibration

Calibration is the process of assigning affiliation scores to the set of case and antecedent variables and is a prerequisite for qualitative comparative analysis. The calibration method proposed by Ragin was followed with reference to relevant studies in the discipline and cross-disciplines [59]. The direct calibration method was used to calibrate the raw data, and the 95%, 50%, and 5% quantile values of the outcome variable and the antecedent variable were set as three anchors of full affiliation, crossover point, and full no affiliation, respectively.

### 4.2 Necessary conditions analysis

The necessity analysis of a single condition is an important step before conducting the condition grouping analysis to determine whether there is a single condition variable that is critical to the development of rural sports. To conduct a comparative test of the analysis results, the effect sizes of the eight antecedent variables were calculated separately using two different estimation methods, the regression ceiling technique (CR) and the envelope ceiling technique (CE) [60]. In the analyzed results, the value of the effect size d is a value in the range of 0–1. It indicates the extent to which the antecedent conditioning variables are necessary for the outcome to occur, and a smaller value of the effect size represents a smaller effect. According to the research proposal, the necessary conditions calculated using the NCA method must satisfy two conditions at the same time: (1) the Effect size is not less than 0.1 and (2) the results of Monte Carlo simulation replacement test show that the effect size is significant.

As shown in Table 4, except for investment in social capital and construction of sports facilities, the P values of the remaining six conditional variables are all greater than 0.01 and do not reach the level of significance. They therefore do not represent the necessary conditions for the development of rural sports. Although the effect size of social capital use and sports field construction is greater than 0.1 and the p-value is significant, they also do not represent a necessary condition for rural sports development, since their accuracy is less than 95%. In summary, from the results of the analysis of condition variables using the NCA method, none of the eight condition variables of rural sports development individually represent a necessary condition for the high-quality development of rural sports.

**Table 4:** Analysis of the necessary conditions for the development of rural sports.

	fdh	Accuracy (%)	Ceiling zone	Scope	Effect size	P-value
Populations	CR	93.3	0.077	0.87	0.088	0.222
	CE	100	0.106	0.87	0.121	0.173
Economic development	CR	100	0.000	0.91	0.000	1.000
	CE	100	0.000	0.91	0.000	1.000
Infrastructure construction	CR	93.3	0.042	0.91	0.046	0.519
	CE	100	0.049	0.91	0.054	0.346
Agricultural production	CR	93.3	0.103	0.90	0.114	0.321
	CE	100	0.119	0.90	0.132	0.176
Grassroots organizations	CR	93.3	0.145	0.91	0.159	0.171
	CE	100	0.181	0.91	0.198	0.037
National financial input	CR	86.7	0.191	0.90	0.212	0.036
	CE	100	0.211	0.90	0.234	0.004
Social capital investment	CR	83.3	0.260	0.88	0.294	0.000
	CE	100	0.272	0.88	0.307	0.000
Rural sports fields	CR	83.3	0.304	0.91	0.334	0.000
	CE	100	0.285	0.91	0.312	0.000

Table 5 shows the results of the bottleneck-level analysis for rural sports development. The bottleneck level is the value of the level that must be met to reach a level within the maximum observed range of the outcome and the maximum observed range of the antecedent conditions, as shown in Table 5, to achieve 100 percent level of rural sports development. Then it is necessary to maintain the main force, the working-age population, at a level of 41.9%, a level of infrastructure construction of 11.2%, a level of modernization of agricultural production of 40.2%, a level of basic Organizational guarantees of 75.6% to be used, 82.5% social capital participation and 94.8% sports field construction.

**Table 5:** Bottleneck-level analysis of conditional variables.

	Populations	Economic development	Infrastructure construction	Agricultural production	Grassroots organizations	National financial input	Social capital investment	Rural sports fields
0	NN	NN	NN	NN	NN	NN	NN	NN
10	NN	NN	NN	NN	NN	NN	NN	NN
20	NN	NN	0.2	NN	NN	NN	NN	NN
30	NN	NN	1.6	NN	NN	NN	1.5	0.6
40	NN	NN	3.0	NN	NN	NN	13.1	14.0
50	NN	NN	4.4	4.9	NN	NN	24.7	27.5
60	2.0	NN	5.7	12.0	3.6	1.7	36.2	41.0
70	12.0	NN	7.1	19.0	21.6	27.3	47.8	54.4
80	22.0	NN	8.5	26.1	39.6	53.0	59.4	67.9
90	31.9	NN	9.9	33.1	57.6	78.6	70.9	81.3
100	41.9	NN	11.2	40.2	75.6	NA	82.5	94.8

### 4.3 Conditional grouping analysis

The condition variables and outcome variables of rural sports development were imported into fsQCA3.0 software for condition grouping analysis. Based on the actual situation of the study cases, the consistency threshold was set at 0.9 and the PRI threshold was set at 0.75. Combined with the sample size of cases, the frequency threshold was set at 1 to ensure that it could cover the 30 cases of the study. In conducting standardized analyzes for high-quality development of rural sports. Given the spatial heterogeneity of rural sports development in China and the actual situation of large differences in resource endowment and development environment, there is no uniform necessity condition for rural sports development. That’s why we don’t give direction and choose “present or absent”. Finally, the three solutions of simple, medium and complex rural sports development are identified based on the standardized analysis.

As shown in Table 6, there are four conditional groupings that can improve the level of rural sports development in China. The high level of consistency indicates that all four grouping paths represent sufficient conditions for high-quality rural sports development and better represent the characteristics of the high-quality rural sports development case. Among them, the overall consistency is 0.965 and the coverage is 0.629, which means that 96.5% of all rural sports development cases that meet the 4 grouping paths have a high level of provincial rural sports development. And it can explain about 62.9 percent of cases of high-quality rural sports development, which has good explanatory power.

**Table 6:** Rural sports quality development grouping.

Variables	Path 1	Path 2	Path 3	Path 4
Populations	⚫	⚫	Ⓧ	Ⓧ
Economic development	●		●	ⓧ
Infrastructure construction	⚫	⚫	Ⓧ	Ⓧ
Agricultural production		●	ⓧ	ⓧ
Grassroots organizations	ⓧ	●	ⓧ	●
National financial input	●	●	●	●
Social capital investment	●	●	●	●
Rural sports fields	⚫	●	●	●
Consistency	0.978	0.957	0.968	0.967
Raw coverage	0.306	0.513	0.209	0.290
Unique coverage	0.042	0.184	0.009	0.033
Solution consistency	0.965			
Solution coverage	0.629			

Note: ● = core condition present; Ⓧ = core condition missing; ⚫ = borderline condition present; ⓧ = borderline condition missing; blanks indicate may or may not be present

### 4.4 Robustness test

Due to the lack of research data on rural sports in other years, adjustment of PRI consistency and exclusion of special cases were carried out to conduct the robustness test of the analysis results. The PRI consistency is adjusted from 0.75 to 0.80, and the group inferences generated after the adjustment are essentially the same as those before the adjustment. Considering the possible differences between municipalities directly under the central government and other provinces in terms of resource factors, the grouping conclusions generated after excluding the four cities of Beijing, Tianjin and Chongqing are also consistent with the previous conclusions. This suggests that the grouping conclusions are robust.

## 5. Discussion

The results of fsQCA analyzes show that there are four grouping paths that can promote the high-level development of rural sports in China, suggesting that the high-level development of rural sports in China can be realized through various paths. In addition, the resource endowment with the ensemble of state financial contributions, social capital participation and sports facilities and facilities is the central driving factor for the development of rural sports in China. When analyzing the grouping of conditions leading to low-quality development of rural sports, there are four groupings of conditions leading to low-quality development of rural sports in China. In the discussion section, typical cases of high level of rural sports are analyzed in more detail to highlight the specific differences and characteristics of rural sports development in different regions of China.

### 5.1 Grouping paths for high level development of rural sports

There are four conditional groupings that can promote a high level of rural sports in China. Combined with the grouping characteristics, the pathways are categorized into three configurations: (1) led by economic development and jointly controlled by state and social capital, (2) resource endowment promoted by modernization of agricultural production, and (3) grassroots organization supported by resource endowment advantages.

#### 5.1.1 Led by economic development and jointly controlled by state and social capital

This configuration mainly covers the two grouping paths of path 1 and path 3 in Table 6. The higher income of rural residents, the high attention of the government and the active participation of social capital are the key driving factors for the development of rural sports, forming a model jointly supported by the state and social capital and supported by the high income of rural residents. This shows that even without grassroots organizations, a better economic level of rural areas, coupled with the government’s financial investment and high social capital participation, can still achieve high-quality rural sports development.

Economically developed areas such as Jiangsu and Zhejiang provinces are typical examples of this grouping. Among other things, the precise rural development and reform system is an important reason for the rising income level of rural residents. For example, more than 99% of the villages in Jiangsu Province have completed the reform of the rural collective property rights system, and the rate of confirmation, registration and issuance of land contract management rights certificates has reached 98.4%. Zhejiang Province, on the other hand, has embarked on rural collective farm reform to continuously improve the income of rural residents, and in 2022, 98.9% of administrative villages in the province reached 200,000 yuan of the total collective farm income, more than 100,000-yuan farm income. On the other hand, the active participation of social actors has promoted the flow of public sports resources between urban and rural areas, and urban and rural sports development resources are increasingly aligned. Since the 14th Five-Year Plan, Zhejiang Province has taken the lead in realizing the urban-rural integration of the "fifteen-minute fitness circuit", and through investment in social capital, more than 120 national fitness activity centers have been established in the city (on the street) built and national fitness centers in central villages and modernized 500 prosperous sports villages. As a nationwide demonstration area of the public sports service system, the national fitness facilities in Jiangsu Province continue to improve, forming a five-tier network of sports facilities, covering urban and rural areas, fully functioning provinces, cities, counties and villages, and sports facilities in administrative villages to ensure complete coverage and continue to expand into concentrated farmer residential areas and larger natural villages.

In contrast to path 1, in path 3 the construction of sports facilities appears as a core condition, while the main sports force and infrastructure construction are missing as core conditions. Among them, Heilongjiang, Liaoning, Tianjin and other provinces with better development of ice and snow sports and light industry are typical cases of Path 3. Path 3 shows that the lack of sports key workers and the lack of rural infrastructure construction are not a cause too severe restriction of the rural sports development. The reason for this is that sophisticated ice and snow sports have become catalysts for the development of rural sports. As part of the country’s efforts to increase the supply of ice and snow tourism products, promote the high-quality development of ice and snow tourism, and better meet the public’s demand for ice and snow tourism and consumption, public participation Ice and snow sports experiences have greatly increased and stimulated consumption. A series of traditional folk ice and snow sports activities, such as ice skating and skiing, have also promoted the development of the rural sports industry, the income channels of farmers have expanded, and their income level has continued to rise. In addition, the large influx of ice and snow sports enthusiasts and tourists has offset the limiting effects of population loss and population aging on rural sports development. However, rapid population loss and aging are still urgent problems for rural sports development in the future, and how to reduce the loss of the backbone of rural sports development, which is mainly young adults, should be one of the topics for future research.

#### 5.1.2 Resource endowment promoted by modernization of agricultural production

Path 2 is the typical representative of this configuration and constitutes a rural sports development model based on the modernization of agricultural production, the participation of the state and social capital, and the construction of rural sports facilities. Henan, Sichuan, Hebei, Hubei, Shandong and other large agricultural and rural provinces where primary industry development dominates are typical cases of this configuration.

Path 2 shows that the modernization of agricultural production methods is an important feature of the rural sports development model in the above regions. In this part of the country, the modernization of agricultural production methods has a driving effect on the development of rural sports, which is reflected in the fact that it frees farmers from heavy labor activities, improves the efficiency of agricultural production, and at the same time increases the income of rural residents and creates the Requirements for the participation of rural residents in sports and physical education. For example, in Henan Province, the comprehensive mechanization rate of plowing, planting and harvesting of major crops has reached more than 85%, and grain production has exceeded 65 billion kilograms in four consecutive years; In Sichuan Province, 7.4 million hectares of high-quality farmland have been cultivated, and the total output of agricultural machinery has reached 47.5 million kilowatts. In Hubei Province, 31 modern agricultural industrial parks were created, and 36,000 bases were built for the modernization of the agricultural industry. and 20 provincial-level modern agriculture demonstration zones have been established in Hebei Province. The total output value of the agricultural product processing industry reached 4.5 trillion, ranking among the top 5 in China. With the great attention of the government and the active participation of social capital, the construction of rural sports venues and facilities in the above-mentioned areas has also become more and more precise. Sichuan has built a total of 5,026 village-level sports and fitness projects, covering an area of 1,273,212 square meters of rural sports and fitness facilities, while Henan has 3,437 and 3,691,273 square meters, Hubei has 1,237 and 764,312 square meters, and Hebei has 2,168 and 809,538, respectively square meters and Shandong has 3,001 and 7,964.24 square meters respectively. As a typical representative of China’s large agricultural and rural provinces, the larger rural population base is the backbone and inexhaustible impetus for the development of rural sports, and the role of rural youth should be strengthened in promoting the development of rural sports. In path 2, the original coverage of 0.513 shows that rural sports in most areas of China are currently dominated by the resource-based development method of agricultural production modernization.

#### 5.1.3 Grassroots organization supported by resource endowment advantages

In Path 4, with state financial participation, social capital participation and sports facility construction as key driving elements and grassroots organization guarantees as secondary conditions, this is the main representative of this configuration. Gansu, Ningxia, Shaanxi and other western regions are typical cases of this configuration. This configuration shows that even in the absence of the main force of young adults and the incomplete construction of rural infrastructure, the high attention of the government, the active participation of social capital and the optimal facilities of sports and fitness venues can still ensure better development of rural sports.

Gansu, Ningxia, Shaanxi and other provinces are located in the western inland, and the lack of major industries, lack of transportation and poor natural environment are the main reasons for their slow socio-economic development. The lack of self-development ability means that the development of rural sports in Gansu, Ningxia and Shaanxi relies on the support and support of the state and social institutions. According to statistics, in 2021, the government invested 31.2 million in financial resources and 32.47 million in social sources to promote the development of rural sports in Gansu Province. With a large amount of development resources invested by the government and society, Gansu Province has built a total of 1,268 village-level farmer fitness projects with a coverage rate of 85%, 1,655 fitness trails, and 503 townships and community sports and fitness centers. Combined with reality, it is not difficult to find that the advantage of resource endowment within the framework of grassroots organizations becomes the epitome of rural sports development in most economically underdeveloped regions of China. This development approach also reveals the core of the development of rural sports in China, namely the inertia of the government’s historical development path in providing public services in rural sports of a single, and the government’s financial pressure continues to increase. At the same time, it is difficult to control the level of improve rural sports development.

### 5.2 Grouping paths for non-high-level development of rural sports

In order to further explore the mechanism of various driving factors of rural sports development, the group paths leading to low-quality rural sports development are further examined, and four group paths of low-quality rural sports development are presented Table 7. Path 1 shows that it is difficult to realize high-quality rural sports development in a development environment where the main force of rural sports development is missing, the level of economic development is low, agricultural production methods are traditional, and government financial investment is low inadequate, and the construction of sports facilities is inadequate. Provinces such as Qinghai and Shanxi are typical cases of this configuration. Path 2 shows that even with better grassroots organizations and institutional protections, inadequate construction of sports facilities and lower levels of economic development can affect the level and quality of rural sports development. Guangxi and Yunnan are typical representatives of this configuration. Finally, Path 3 and Path 4 show that in the case of failed modernization of agricultural production and insufficient state financing, the level of rural sports development will not be high even with better construction of rural infrastructure. The provinces of Fujian, Guangdong and Jiangxi are typical cases of this group.

**Table 7:** Rural sports nonquality development grouping.

Variables	Path 1	Path 2	Path 3	Path 4
Populations	Ⓧ	⚫	Ⓧ	⚫
Economic development	Ⓧ	ⓧ	⚫	⚫
Infrastructure construction	ⓧ	⚫	●	●
Agricultural production	Ⓧ	⚫	Ⓧ	Ⓧ
Grassroots organizations		●	ⓧ	⚫
National financial input	Ⓧ	⚫	Ⓧ	Ⓧ
Social capital investment	ⓧ		⚫	⚫
Rural sports fields	Ⓧ	Ⓧ	ⓧ	⚫
Consistency	0.963	0.988	0.996	0.993
Raw coverage	0.441	0.267	0.162	0.191
Unique coverage	0.268	0.086	0.020	0.031
Solution consistency	0.972			
Solution coverage	0.603			

Note: ⚫ = core condition present; Ⓧ = core condition missing; ● = borderline condition present; ⓧ = borderline condition missing; blanks indicate may or may not be present

By comparing the grouping path to high-quality rural sports development, it is found that although the modernization of agricultural production is not the basic requirement for the high-quality development of rural sports, the tradition of However, agricultural production methods are the core condition that leads to low-quality development of rural sports, which reveals an asymmetric causal relationship of rural sports development. In addition, through the combination of the four grouping paths of low-quality rural sports development, the development of rural sports has the characteristics of dependence on agricultural production methods, government financing and the construction of rural sports venues and facilities. That is, if these three conditions are not met, the level of rural sports development will be limited regardless of the level of rural infrastructure construction and economic development.

### 5.3 Typical case analysis of rural sports development

**Fig 3:**
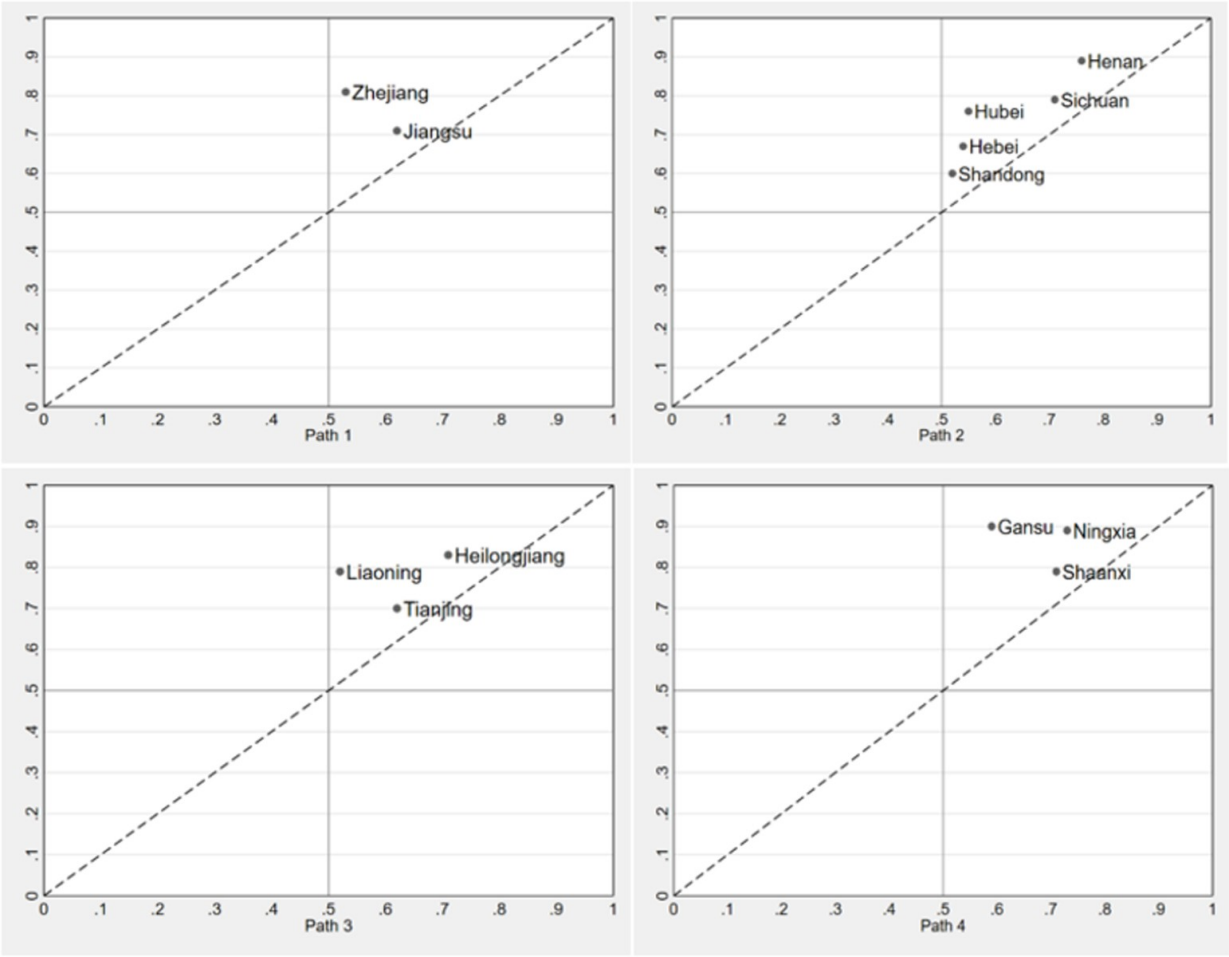
Typical cases of high-quality development of rural sport.

As shown in Fig 3, The typical cases of Path 1 are mainly concentrated in the economically developed east coast r0egion, which includes Zhejiang and Jiangsu provinces. The higher income of the rural population is an important guarantee for the high-quality development of rural sports, while the precise construction of rural sports facilities and rural infrastructure plays a supporting role. The lack of grassroots sports organizations reflects that rural residents in these regions have a high awareness of physical activity and that the development of rural sports does not necessarily depend on rural grassroots sports organizations. Typical cases of Path 2 are mainly concentrated in the central and western regions and include large agricultural and rural provinces such as Henan, Sichuan, Hubei, Hebei and Shandong. In a development environment dominated by primary industry, the modernization of agricultural production fully realizes its role in the development of rural sports in the above-mentioned regions. The emergence of the rural sports development model, which is mainly characterized by the modernization of agricultural production, is a typical resource-based model of modernization of agricultural production. The typical cases of Path 3 are mainly concentrated in the northeastern regions such as Heilongjiang, Liaoning and Tianjin. The rural sports development patterns of the above regions show that even in a development environment where the main force of rural sports is missing, aging is increasing, and rural infrastructure needs to be improved, a higher level of economic development, greater government attention, Through the active with the participation of social capital and ideal sports venues and facilities, high-quality development of rural sports can also be realized. The developed ice and snow sports tourism business offsets the limiting impact of population decline and aging on rural sports development. Path 4 focuses mainly on western provinces such as Ningxia, Gansu and Shaanxi, where improved rural sports venues and facilities, highly valued by the government and actively contributing to social capital, have become the key drivers of rural sports development.

## 6. Conclusion and implications

Compared with previous studies, this study found that there are large differences in the level of rural sports development in different provinces in China. The level of rural sports development tends to be higher in economically developed areas, such as Beijing, Tianjin, Zhejiang, Jiangsu and other provinces, whose super-efficiency values for rural sports development are 1.569, 1.461, 1.153, 1.208, etc. This is in high consistency with the results of previous studies. In addition, the driving role of the construction of sports venues and facilities in rural areas and the state’s financial input on rural sports development is reflected in this study, and it also verifies the findings of previous studies. However, the efficiency of rural sports development is still better in certain economically underdeveloped areas, such as Ningxia, Gansu, Inner Mongolia, Hainan and other provinces. The reason for this is that the state attaches great importance to the development of rural sports in economically backward areas, and the large amount of financial investment and the construction of sports venues and facilities have maintained the high level of rural sports development in the above areas. Population size in rural areas does not play a significant role in driving rural sports development, while agricultural production methods play a larger role. These findings reflect the differences between this study and previous studies. Overall, this study enriches and refines previous research at both the theoretical and practical levels.

In the context of the rural revitalization strategy and the era of a healthy China, the development of rural sports has become an important strategic support for the comprehensive revitalization of villages. The level and quality of rural sports development play an important role in promoting the development of rural areas as well as building a sports center. The existing literature mainly examines the realistic dilemma and development path of rural sports in China, paying less attention to the linkage mechanism between various influencing factors of rural sports development. On this basis, this study introduces the theory of spatial heterogeneity and builds a theoretical model of rural sports development based on development object, development environment and resource endowment. 30 provinces in China are selected as study cases, and the DEA-SBM model and qualitative comparative analysis with fuzzy sets are comprehensively applied. Accurately identify the driving elements of rural sports development in China and explore the synergy between different driving elements to create mechanisms. Construct multiple grouping paths to improve the development level of rural sports in China and reveal the characteristics and differences of rural sports development in different regions of China. Providing guidance and practical suggestions for comprehensively improving the development level of rural sports in China.

### 6.1 conclusion

The conclusions of this study are as follows. (1) There is significant spatial heterogeneity in the development of rural sports in China, and there are large differences in the level of rural sports development in different regions. Among them, Beijing, Tianjin, Zhejiang, Jiangsu and other economically developed provinces have a better level of rural sports development, with development super efficiency scores of 1.569, 1.461, 1.153 and 1.208, respectively. The development efficiency of rural sports in economically backward regions such as Ningxia, Inner Mongolia and Gansu is still high, which is probably related to the high national financial contribution. (2) There are three main models for rural sports development in China, including three configurations: state-social capital jointly driven by economic development, resource endowment driven by the modernization of agricultural production, and grassroots organization driven by the Advantage of resources supported equipment. Resource provision is the key driving factor for the development of rural sports, with government funding, participation in social capital and sports venues and facilities forming the basis. (3) There are obvious differences in rural sports development patterns in different regions. The high importance attached by the government and the active participation of social capital, complemented by higher income of rural residents and solid rural infrastructure development, are important features of rural sports development in Jiangsu and Zhejiang. The modernization of agricultural production methods is a typical feature of rural sports development in agricultural provinces with good primary industry development, such as Henan, Sichuan, Hubei, Hebei and Shandong, in addition to their rich resource endowment. Heilongjiang, Liaoning, Tianjin and other provinces, on the other hand, promote rural sports development with ice and snow sports. In Gansu, Ningxia, Shaanxi and other economically underdeveloped regions in the West, the construction of rural sports venues and facilities, improved by the government’s great attention and the help of social institutions, is the main reason for improving the level of rural sports development in the region.

### 6.2 Suggestion

Based on the conclusions of the study, the following practical recommendations are made. (1) Combine resource equipment and refine the top-level design of rural sports development. Regions with better rural sports development can continuously optimize the rural sports development model according to the group structure and reduce the redundancy of resource use in the development process. For less economically developed areas, the rural sports development pattern should be actively investigated by government, market and social provision. (2) Promote the modernization of agricultural production methods, implement industrial feedback in agriculture, and increase the productivity of rural residents. The mechanization of agricultural production methods improves the efficiency of agricultural production while increasing the income of rural residents, so that rural residents have time and energy to participate in sports and exercise activities. (3) Improve the construction of rural infrastructure, taking into account the practical needs of rural residents for physical activity, and continuously improve the construction of rural sports facilities. Give full play to the role of the environment in transforming people and raise rural residents’ awareness of physical exercise to realize the role of sports in nurturing people, educating the intellect and strengthening the body. (4) Continuously promote the construction of digital infrastructure in rural areas and expand channels for rural sports advertising, promotion and advice using the Internet and technical means. Together with Internet short video platforms, rural sports stars are created and the star effect is used to increase the quality and effectiveness of rural sports development.

### 6.3 Implication

This study has important theoretical significance and practical significance. It can provide theoretical guidelines and ideas for the design and adjustment of China’s rural sports development strategy, to effectively improve the level and quality of China’s rural sports development and promote the comprehensive rural revitalization.

In terms of theoretical meaning. This study innovatively creates a theoretical model of rural sports development based on spatial heterogeneity theory. The development model covers the three aspects of the development object, development environment and resource endowment, as well as the driving role of various aspects for rural sports development is reflected in this study. The development model can be further applied and tested in follow-up studies. In addition, this study innovatively introduces the theory of spatial heterogeneity to analyze the geographical differences and characteristics of rural sports development in different regions of China. The spatial heterogeneity of rural sports development is tested. In combination with the Fuzzy Set qualitative comparative analysis method, the complex causal relationship between rural sports development is clarified. Using the DEA-SBM model, the efficiency of rural sports development in China is evaluated and the current situation of rural sports development is analyzed. Overall, this study theoretically complements and extends the existing research literature and introduces new perspectives and methods for studying rural sports development in China.

In terms of practical significance. This study evaluates the efficiency of rural sports development in China, accurately identifies the key driving factors of rural sports development in China, and reveals the geographical differences that exist in rural sports development. Typical cases of high-level development of rural sports are also analyzed in detail. In addition, the theoretical model and grouping path constructed in this study can serve as a guide for the development of rural sports and help other provinces make decisions in raising the level of rural sports development. This will make the strategic design and adjustment of rural sports development more scientific, effective and accurate, thereby improving the efficiency of rural sports development and avoiding the waste of resources.

### 6.4 Limitations and prospects

This study has the following limitations: (1) Due to the number of cases, although this study integrated as many influencing factors of rural sports development as possible, not all of them were covered due to the complexity and variability of rural sports development, and future research can select other influencing factors to explore the mechanism of the role of different influencing factors on rural sports development. (2) Due to the availability of data, the data obtained in this paper were static data and failed to represent the dynamic evolution of influencing factors over time. Future research can use the time series QCA method to explore the trajectory of rural sports development in different regions based on time panel data to further improve the level of rural sports development.

## Supporting information

S1 Data (XLSX)
